# Reported association of air pollution and suicide rate could be confounded

**DOI:** 10.1186/s12940-017-0219-3

**Published:** 2017-02-28

**Authors:** R. Afshari

**Affiliations:** 1 BC Centre for Disease Control, 655 West 12th Avenue, Vancouver, BC V5Z 4R4 Canada; 2 Occupational and Environmental Health Division, School of Population and Public Health, University of British Columbia, Vancouver, Canada

**Keywords:** Air pollution, Suicide, Underreporting, Selection bias

## Abstract

A statistical association between ambient air pollution and suicide mortality has been recently reported in Environmental Health, which seems not to be scientifically supported by their data.

In this article, very low (unrealistic) suicide rate is reported, which is subjected to selection bias. Their justification is also flawed as high exposure to ambient air pollution in rural areas is lower as compared to urban residents. Weekends, holidays, time of death … are also both air pollution and suicide rate related. Reported statistical association of air pollution and suicide in this study is heavily confound.

Please see article under discussion: https://ehjournal.biomedcentral.com/articles/10.1186/s12940-016-0177-1.

Dear Editor,

I have read with interest your recent publication on association between ambient air pollution and suicide mortality [1]. Their conclusion does not seem to be epidemiologically supported.

The total rate of suicide in East Asian countries such as Japan and South Korea is relatively higher (19 × 10^−5^ and 17 × 10^−5^) than the rest of the world [2]. China, however, reports lower rates.

According to the Bulletin of the World Health Organization, suicide in China accounts for about a quarter of all suicides worldwide in the past decades [3]. High rates have also been reported in the recent years including 14.7 to 9.1 × 10^−5^ 2006-2012 [4], 46 × 10^−5^ [5], and 34.5 × 10^−5^ (elderly suicide rate) in this country [6].

Authors reported a total of 1 550 registered suicide deaths in Guangzhou with a population of 7.7 million permanent residents (i.e. 60.8% of population of Guangzhou) between 2003 and 2012 [1]. Taking these values, the suicide rate would be 2.2 × 10^−5^, which is far lower than the lowest reported rates and subjected to underreporting. The extent of which seems to be large enough to reasonably question their findings.

The rate of suicide underreporting for different sexes, age groups, sexuality tendencies and types of suicide are diverse [7–10], which authors superficially mentioned. In addition to subpopulations, there are systematic reasons for underreporting including low accuracy in determining the underlying causes of deaths [11], inaccurate collection and coding (misclassifying of suicides as injuries) that are problematic for data stakeholders [12], stigma [13] and high standards of proof [12]. Majority of these reasons, for example, are different for urban population with high exposure to ambient air pollution and rural residents in which air pollution is low. As a result under reporting in this study is subjected to selection bias. Weekends, holidays, time of death … are also both air pollution and suicide rate related, and confound their findings to a further extent.

Reported association between air pollution and suicide is at best questionable. Findings should be treated with caution.
